# 02. Beyond B Antigen Coverage: The Potential of the 4CMenB Vaccine for Cross-protection Against Pathogenic *Neisseria* Infections

**DOI:** 10.1093/ofid/ofab466.205

**Published:** 2021-12-04

**Authors:** Yara Ruiz Garcia, Woo-Yun Sohn, Mariagrazia Pizza, Rafik Bekkat-Berkani

**Affiliations:** 1 GSK, Rockville, Maryland; 2 GSK Vaccines, Siena, Toscana, Italy

## Abstract

**Background:**

Two human pathogenic *Neisseria* species exist: *N. meningitidis* (*Nm*) and *N. gonorrhoeae* (*Ng*). Although causing disparate clinical syndromes, invasive meningococcal disease (IMD) and gonorrhea, they are genetically similar and share key protein antigens. The 4CMenB vaccine, licensed against meningococcal B disease, comprises 4 antigenic components (factor H binding protein (fHbp), variant 1.1, subfamily B; Neisseria heparin binding antigen (NHBA) peptide 2; Neisserial adhesin A (NadA) variant 3; and Porin A (PorA) P1.4), and potentially protects against non-B invasive meningococcal and gonococcal strains. In this review, we summarize the similarities between these antigens and those in *Nm* serogroups A, C, W, X and Y and *Ng*.

**Methods:**

Published data in humans were analyzed to conduct a narrative literature review of the potential extent of meningococcal vaccine-induced protection against non-B meningococcal strains and *Ng*. Techniques applied to indirectly measure this effect are based on genotype-phenotype modelling, strain coverage, bactericidal killing and direct impact on disease reduction.

**Results:**

Data were identified from countries in America, Europe, Africa and Oceania. The genes encoding for fHbp and NHBA are also present in strains belonging to the five non-B serogroups, while NadA is present in several strains of serogroups C, W and Y, and PorA P1.4 mainly in serogroup W. At the genome level, *Ng* and *Nm* share up to 90% homology. Most of the outer membrane vesicle antigens, like PilQ, Omp85 (BamA), NspA, MtrE, MetQ, LbpA, PorB, FetA, OpcA and NHBA, are highly conserved in *Ng*. In addition, a synergistic effect might enhance immunogenicity against non-B serogroups as shown against serogroup B.

**Conclusion:**

4CMenB components are present and conserved in several *Ng* and *Nm* strains. Recent results demonstrate that 4CMenB reduces MenW disease incidence in infants and might generate cross-protection against other non-B serogroups. In addition, 4CMenB has been proven to be effective in reducing gonococcal infections in adolescents. Research on future genomic and proteomic characterizations of IMD and gonorrhea strains will provide information on the molecular basis of the underlying broad strain coverage, while informing decisions regarding prevention and immunization programmes.

**Disclosures:**

**Yara Ruiz Garcia, MSc, PhD**, **GSK group of companies** (Employee) **Woo-Yun Sohn, MD**, **GSK group of companies** (Employee, Shareholder) **Mariagrazia Pizza, Biological Sciences, PhD**, **GSK group of companies** (Employee, Shareholder) **Rafik Bekkat-Berkani, M.D**, **GSK group of companies** (Employee, Shareholder)

